# Treatment Strategies for Chronic Coronary Heart Disease with Left Ventricular Systolic Dysfunction or Preserved Ejection Fraction—A Systematic Review and Meta-Analysis

**DOI:** 10.3390/pathophysiology30040046

**Published:** 2023-12-18

**Authors:** Elena Zelikovna Golukhova, Inessa Viktorovna Slivneva, Olga Sergeevna Kozlova, Bektur Shukurbekovich Berdibekov, Ivan Ivanovich Skopin, Vadim Yuryevich Merzlyakov, Renat Kamilyevich Baichurin, Igor Yuryevich Sigaev, Milena Abrekovna Keren, Mikhail Durmishkhanovich Alshibaya, Damir Ildarovich Marapov, Milena Artemovna Arzumanyan

**Affiliations:** 1 A.N. Bakulev National Medical Scientific Center for Cardiovascular Surgery, 121552 Moscow, Russia; egolukhova@bakulev.ru; 2 Department of Cardiovascular and Comorbid Pathology, A.N. Bakulev National Medical Scientific Center for Cardiovascular Surgery, 121552 Moscow, Russia; oskozlova@bakulev.ru (O.S.K.); rkbaichurin@bakulev.ru (R.K.B.); maarzumanyan@bakulev.ru (M.A.A.); 3 Department of Non-Invasive Arrhythmology and Surgical Treatment of Combined Pathology, A.N. Bakulev National Medical Scientific Center for Cardiovascular Surgery, 121552 Moscow, Russia; bsberdibekov@bakulev.ru; 4 Department of Reconstructive Surgery of Heart Valves and Coronary Arteries, A.N. Bakulev National Medical Research Center for Cardiovascular Surgery, 121552 Moscow, Russia; iiskopin@bakulev.ru; 5 Department of Surgical Treatment of Ischemic Heart Disease and Minimally Invasive Coronary Surgery, A.N. Bakulev National Medical Research Center for Cardiovascular Surgery, 121552 Moscow, Russia; vymerzlyakov@bakulev.ru; 6 Department of Surgical Treatment of Coronary and Great Arteries Combined Diseases, A.N. Bakulev National Medical Scientific Center for Cardiovascular Surgery, 121552 Moscow, Russia; iysigaev@bakulev.ru (I.Y.S.); makeren@bakulev.ru (M.A.K.); 7 Department of Surgical Treatment of Ischemic Heart Disease, A.N. Bakulev National Medical Scientific Center for Cardiovascular Surgery, 121552 Moscow, Russia; mdalshibaya@bakulev.ru; 8 Department of Public Health, Economics and Health Care Management, Kazan State Medical Academy—Branch Campus of the Federal State Budgetary Educational Institution of Further Professional Education, Russian Medical Academy of Continuous Professional Education, 420012 Kazan, Russia; damirov@list.ru

**Keywords:** chronic coronary heart disease, left ventricular function, systolic dysfunction, revascularization, optimal medical therapy

## Abstract

In this meta-analysis, we examine the advantages of invasive strategies for patients diagnosed with chronic coronary heart disease (CHD) and preserved left ventricular (LV) function, as well as those with significant LV systolic dysfunction (LV ejection fraction (EF) < 45%). Material and methods: We conducted a systematic search to identify all randomized trials directly comparing invasive strategies with optimal medical therapy (OMT) in patients diagnosed with chronic CHD. Data from these trials were pooled using a random-effects meta-analysis. The primary outcome assessed was the all-cause mortality, while secondary endpoints included cardiovascular (CV) death, stroke, myocardial infarction (MI), and unplanned revascularization. This study was designed to assess the benefits of both invasive strategies and OMT in patients with preserved LV function and in those with LV systolic dysfunction. The statistical analysis of the data was conducted using the Review Manager (RevMan) software, version 5.4.1 (The Cochrane Collaboration, 2020). Results: Twelve randomized studies enrolling 13,912 patients were included in the final analysis. Among the patients with chronic CHD and preserved LV systolic function, revascularization did not demonstrate a reduction in all-cause mortality (8.52% vs. 8.45%, *p* = 0.45), CV death (3.41% vs. 3.62%, *p* = 0.08), or the incidence of MI (9.88% vs. 10.49%, *p* = 0.47). However, the need for unplanned myocardial revascularization was significantly lower in the group following the initial invasive approach compared to patients undergoing OMT (14.75% vs. 25.72%, *p* < 0.001). In contrast, the invasive strategy emerged as the preferred treatment modality for patients with ischemic LV systolic dysfunction. This approach demonstrated lower rates of all-cause mortality (40.61% vs. 46.52%, *p* = 0.004), CV death (28.75% vs. 35.82%, *p* = 0.0004), and MI (8.19% vs. 10.8%, *p* = 0.03). Conclusions: In individuals diagnosed with chronic CHD and preserved LV EF, the initial invasive approach did not demonstrate a clinical advantage over OMT. Conversely, in patients with ischemic LV systolic dysfunction, myocardial revascularization was found to reduce the risks of CV events and enhance the overall outcomes. These findings hold significant clinical relevance for optimizing treatment strategies in patients with chronic CHD, contingent upon myocardial contractility status.

## 1. Introduction

Chronic coronary heart disease (CHD) remains the most common pathology within the circulatory system, leading to high mortality rates despite advancements in diagnostic and treatment techniques. According to the Global Burden of Disease report in 2022, chronic CHD accounted for over 9.4 million deaths worldwide [1]. It is imperative to prioritize the development of proactive preventive measures and optimize the treatment of chronic CHD to mitigate morbidity and prevent complications that could result in adverse outcomes. In the past decade, extensive research efforts have been made to identify the most effective treatment strategies for chronic CHD.

Myocardial revascularization may be considered in patients with left ventricular (LV) systolic dysfunction and appropriate coronary anatomy to improve disease prognosis and alleviate ischemia by addressing the pathophysiological substrate of hibernating myocardium [2,3]. It is noteworthy that patients with LV systolic dysfunction are typically excluded from randomized clinical trials (RCTs) comparing treatment modalities for chronic CHD. Consequently, there is a scarcity of publications focused on studying the optimal strategies for this specific patient group. Hence, the majority of guidelines are based on observational studies and expert opinions [3,4].

When analyzing current data, contradictions arise concerning the assessment of the impact of myocardial revascularization on total and cardiovascular mortality. Meta-analyses by Bangalore S. et al. [5] and Soares A. et al. [6] demonstrated that, in patients with chronic CHD, routine revascularization (coronary artery bypass grafting (CABG) or percutaneous coronary intervention (PCI)) was not associated with improved overall survival but was linked to a lower risk of myocardial infarction (MI) compared to optimal medical therapy (OMT). According to the meta-analysis by Navarese EP et al. [7], coronary revascularization in patients with stable CHD resulted in lower cardiovascular (CV) mortality compared to OMT, and survival improved with increasing follow-up time. However, a meta-analysis by Wolff G. et al. [8] reported improved overall survival in chronic CHD patients with reduced LV ejection fraction (EF) who underwent CABG (OR 0.66, CI 0.61–0.72, *p* < 0.001) or PCI (OR 0.66, CI 0.62–0.85, *p* < 0.001) compared to OMT, suggesting that CABG may have advantages over PCI in this category of patients. Thus, the question of the positive prognostic influence of myocardial revascularization on the survival of patients with chronic CHD remains open. Further investigation is also warranted to study the dependence of the revascularization effect in high-risk patients with LV systolic dysfunction.

Analyzing the existing RCTs on the treatment of chronic CHD in patients with moderate and severe LV systolic dysfunction will enable the assessment of endpoint risks in high-risk individuals. The acquired data will enhance the precision of existing evidence and systematize conclusions based on the presence of LV ischemic dysfunction, which may be applicable in clinical practice.

Therefore, the objective of the analysis was to investigate the benefits of revascularization compared to OMT for chronic CHD, aiming to diminish the risk of CV events and adverse outcomes. This assessment was conducted in both patients with preserved LV function and those with systolic dysfunction (LV EF < 45%).

## 2. The Search Strategies and Study Selection

The study selection algorithm was designed according to the recommendations and reporting guidelines for systematic reviews and meta-analyses (PRISMA) in PubMed and Google Scholar information systems [9]. The study protocol was registered in PROSPERO (CRD42023486600).

With the defined objective in mind, the search for the required data was carried out using two distinct search strategies. One of them involved seeking publications related to the comparison of myocardial revascularization and OMT in patients with chronic CHD and preserved LV systolic function. In the PubMed database, the query keywords used were as follows: ((((((((((chronic coronary artery disease) OR (stable angina)) OR (stable ischemic heart disease)) AND (optimal medical therapy)) OR (conservative therapy)) OR (conservative strategy)) AND (percutaneous coronary intervention)) OR (myocardial revascularization)) OR (invasive strategy)) AND (coronary artery bypass grafting)) OR (CABG). In the Google Scholar system, the following keywords were employed: chronic coronary heart disease, optimal medical therapy, percutaneous coronary intervention, myocardial revascularization, coronary artery bypass grafting, long-term outcomes, major adverse cardiovascular events (MACE), and all-cause mortality.

Another search query was devised to analyze studies comparing revascularization and OMT in patients with LV systolic dysfunction within the context of chronic CHD. In the PubMed database, the following keywords were utilized: (((((((((((left ventricular systolic dysfunction) OR (ischemic cardiomyopathy)) OR (poor left ventricle)) AND (chronic coronary artery disease)) OR (stable ischemic heart disease)) AND (revascularization)) OR (percutaneous coronary intervention)) OR (coronary artery bypass grafting)) AND (medical therapy)) OR (optimal medical therapy)) OR (conservative therapy)) OR (conservative strategy). For the Google Scholar search, the keywords employed were the following: chronic heart failure with systolic dysfunction, myocardial revascularization, ischemic cardiomyopathy, percutaneous coronary interventions, and coronary artery bypass grafting.

Only publications in English were considered for our analysis. Clinical case reports, expert reviews and opinions, books, abstracts, and meeting protocols were excluded from the meta-analysis. Two investigators independently assessed article titles and publication abstracts for adherence to the inclusion criteria, resolving any disagreements through discussion. The final search for studies was conducted on the 25 September 2023.

## 3. Inclusion/Exclusion Criteria

Studies were considered eligible if they involved a direct comparison of invasive and conservative strategies in chronic CHD and mandated the randomization of patients based on the chosen approach. The inclusion criteria required participants to be older than 18 years. LV systolic dysfunction was determined through echocardiographic studies, with a reduced LV EF below 45%. The follow-up period was set at a minimum of 1 year. An essential condition for the inclusion of publications in the meta-analysis was the adequate presentation of data on clinical endpoints, including all-cause death, CV death, or MI. Considering advancements in revascularization techniques, specifically the widespread use of drug-eluting stents in clinical practice and improvements in OMT, studies conducted after 2007 were analyzed. Non-randomized studies, such as observational studies and registry data, were excluded from the analysis.

## 4. Extraction and Synthesis of Study Data

**Figure 1:**
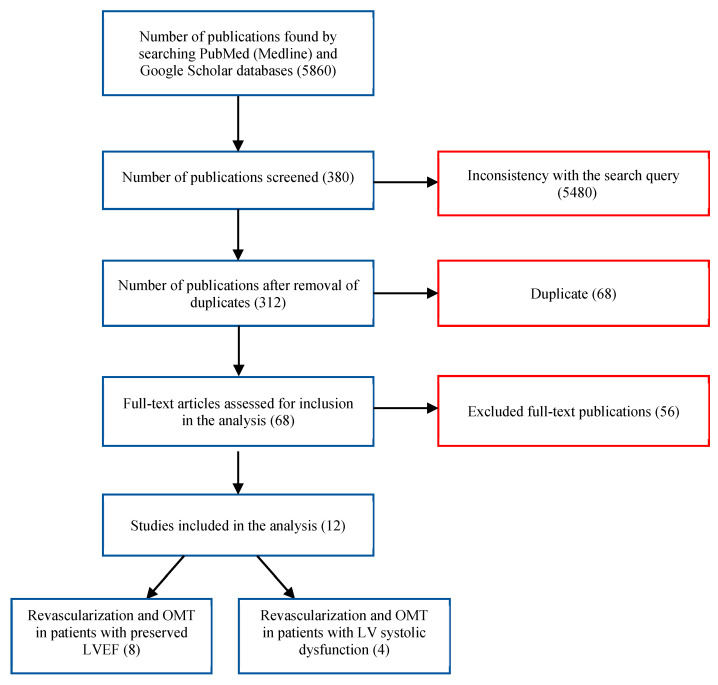
Flowchart depicting the selection process of the studies included in the review.

Initially, the extraction using the aforementioned search queries yielded 2253 publications from PubMed and 3607 results from the Google Scholar information system. Upon analyzing the titles and their abstracts, 380 publications directly aligned with the research objective. Among these findings, 68 duplicates were removed. After a thorough review of the full texts, 56 articles were excluded for not meeting the inclusion criteria. Consequently, twelve studies were selected from the initial search, comprising eight comparative analyses of OMT and revascularization in patients with preserved LV EF and four studies which focused on OMT and revascularization in patients with LV systolic dysfunction. The selection process is illustrated in Figure 1. 

In the presented meta-analysis, the study design involved the separate investigation of patient groups with preserved LV EF and those with LV systolic dysfunction in the ISCHEMIA study (2020). The data on the incidence of endpoints in patients with preserved LV function were obtained by subtracting the results of the subgroup with heart failure and LV dysfunction [10] from the overall results of the ISCHEMIA trial [11].

### Statistical Analysis

The statistical analysis of the data was conducted using the Review Manager (RevMan) software, version 5.4.1 (The Cochrane Collaboration, 2020). The meta-analysis utilized either a random-effects model or a fixed-effects model, employing the inverse variance weighting approach. In cases where the heterogeneity of the study results surpassed 40%, a random-effects model was applied. The results of the meta-analysis were graphically represented using a forest plot. Statistical heterogeneity was assessed using the Q-test and Pearson’s χ^2^ criterion, along with the heterogeneity index I2. The interpretation of statistical heterogeneity followed the recommendations of the Cochrane community: I2 = 0–40% indicated insignificant heterogeneity; 30–60% corresponded to moderate heterogeneity; 50–90% denoted significant heterogeneity; and 75–100% indicated high heterogeneity. Heterogeneity was considered statistically significant at *p* < 0.10 [12].

Dichotomous data were evaluated using a risk ratio (RR) with a 95% confidence interval (CI). Differences at *p* < 0.05 were considered statistically significant. To qualitatively evaluate the presence of systematic bias in meta-analyses comprising five or more studies, funnel plots were constructed. The visual assessment of these plots in Supplementary Figure S1 revealed no significant asymmetry.

Risk of Bias version 2 (RoB2) was employed to assess the potential for systematic error and the level of evidence, as detailed in Supplementary Table S1.

## 5. Results

Twelve RCTs comprising a total of 13,912 patients (7109 randomized to the invasive strategy and 6803 to the conservative therapy) met the search criteria [10,11,13,14,15,16,17,18,19,20,21,22]. The average age of the patients with chronic CHD and preserved LV EF was comparable to the age of those with LV systolic dysfunction (64.7 years and 65.3 years, respectively). Notably, only the study conducted by Won H. in 2016 included elderly patients, aged over 75 years [17].

In the current study, patients with chronic CHD and preserved LV EF exhibited comorbidities such as arterial hypertension (53–82%), cerebrovascular disease (5.4–10.0%), peripheral atherosclerosis (3.4–23.7%), diabetes mellitus (up to 40%), and chronic renal failure (up to 2.7%). The BARI 2D study specifically focused on investigating two treatment strategies in patients with diabetes mellitus and chronic CHD [15].

It is worth noting that patients with LV systolic dysfunction carried a heavier burden of comorbid conditions. They were more prone to cerebrovascular disease (7–17%), diabetes mellitus (up to 48%), and chronic renal failure (up to 8%).

In patients with chronic CHD and preserved LV EF, the primary clinical manifestations of the disease were predominantly at the level of functional class I-II angina. This is evident from various studies: COURAGE—67.5% [13]; JSAP—76.9% [14]; FAME 2—66.9% [19]; and ISCHEMIA—75.5% [11]. In the MASS-II study alone, a significant 78% of patients experienced high functional class angina [16].

It is noteworthy that, in the chronic CHD group with LV systolic dysfunction, the STICH study reported a 58.7% incidence of functional class I-II angina and that 36.5% of patients had no angina attacks [21]. In contrast, the REVIVED-BCIS2 study revealed that 67% of patients showed no symptoms of angina [22].

In the studies involving patients with chronic CHD and preserved LV function, the mean EF ranged from 54.5% to 68.0%, as determined using echocardiography data. In the pooled studies comprising patients with chronic CHD and LV systolic dysfunction, individuals with a mean EF ranging from 27.0% to 45.0% were included. The details regarding the design of individual studies and the baseline characteristics of patients are summarized in Table 1 and Table S2 of the Supplementary Materials.

### 5.1. Chronic CHD with Preserved LV EF

**Figure 2:**
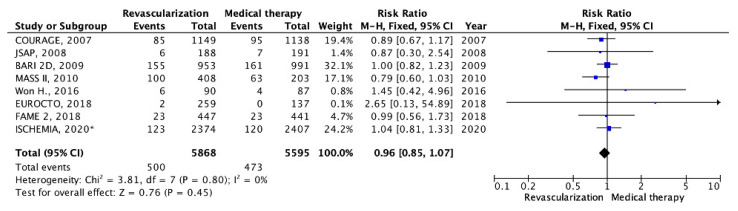
Forest plot of RR for all-cause mortality according to myocardial revascularization compared with optimal medical therapy in patients with chronic coronary heart disease and preserved left ventricular function. “Total events” refers to the sum of all events in the context of all-cause mortality, within each study. Risk ratio for each individual study (blue squares), 95% CI (horizontal lines), black diamond (pooled effect size). * *data obtained by subtracting the results of the studied subgroup with left ventricular systolic dysfunction from the results of the study group with systolic dysfunction in the ISCHEMIA trial (2020),* [11,13,14,15,16,17,18,19].

In all eight RCTs included in the analysis [11,13,14,15,16,17,18,19], data regarding the frequency of all-cause mortality were provided. The total number of events recorded was 500 out of 5868 in the myocardial revascularization group and 473 out of 5595 in the OMT group (Table 2). The average duration of follow-up was 4.2 years. At the conclusion of the follow-up period, there were no statistically significant differences observed in the incidence of all-cause mortality between patients treated with myocardial revascularization and those managed with OMT (8.52% vs. 8.45%; RR: 0.96; 95% CI: 0.85–1.07; *p* = 0.45; I2 = 0%) (Figure 2).

**Figure 3:**
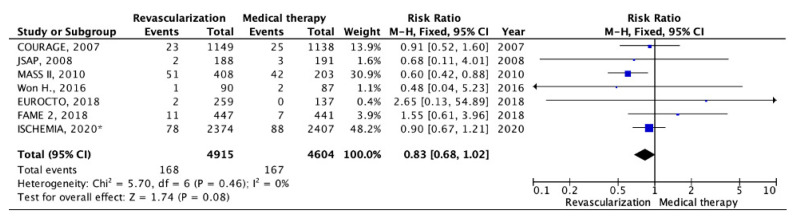
Forest plot of RR for cardiovascular mortality according to myocardial revascularization compared with optimal medical therapy in patients with chronic coronary heart disease and preserved left ventricular function. “Total events” refers to the sum of all events in the context of cardiovascular mortality, within each study. Risk ratio for each individual study (blue squares), 95% CI (horizontal lines), black diamond (pooled effect size). * *data obtained by subtracting the results of the studied subgroup with left ventricular systolic dysfunction from the results of the study group with systolic dysfunction in the ISCHEMIA trial (2020),* [7,8,10,11,12,13,14,16,17,18,19].

Seven RCTs [7,8,10,11,12,13,14,16,17,18,19] provided data on the incidence of cardiovascular mortality. The analysis encompassed information from 4915 patients subjected to myocardial revascularization and 4604 patients managed with OMT. The mean follow-up duration was 4.0 years. Cardiovascular mortality was observed in 168 patients (3.41%) in the myocardial revascularization group and in 167 patients (3.62%) in the OMT group (RR: 0.83; 95% CI: 0.68–1.02; *p* = 0.08; I2 = 0%) (Figure 3).

**Figure 4:**
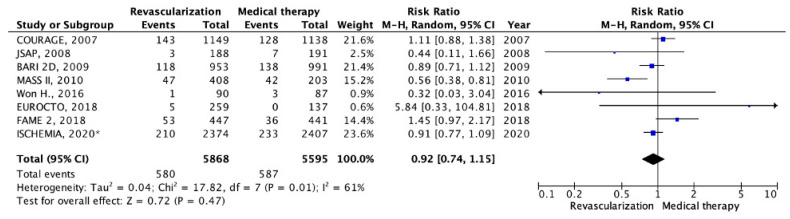
Forest plot of RR for myocardial infarction depending on myocardial revascularization compared with optimal medical therapy in patients with chronic coronary heart disease and preserved left ventricular function. “Total events” refers to the sum of all events in the context of myocardial infarction, within each study. Risk ratio for each individual study (blue squares), 95% CI (horizontal lines), black diamond (pooled effect size). * *data obtained by subtracting the results of the studied subgroup with left ventricular systolic dysfunction from the results of the study group with systolic dysfunction in the ISCHEMIA trial (2020),* [11,13,14,15,16,17,18,19].

The incidence of MI development was reported in eight RCTs [11,13,14,15,16,17,18,19]. In the revascularization group, there were 580 events among 5868 patients, while in the OMT group there were 587 events among 5595 patients. The mean duration of the follow-up period was 4.2 years. The meta-analysis revealed a lower MI frequency in the group of patients undergoing myocardial revascularization compared to those receiving OMT. However, these differences did not reach a statistical significance (9.88% vs. 10.49%; RR: 0.92; 95% CI: 0.74–1.15; *p* = 0.47; I2 = 61%) (Figure 4).

**Figure 5:**
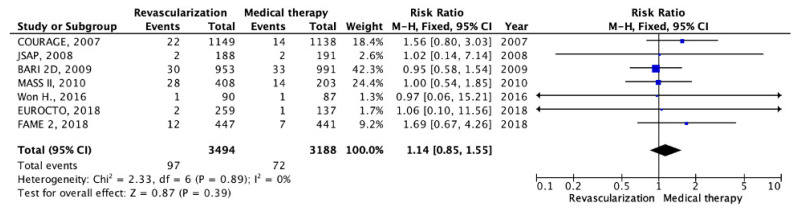
Forest plot of RR for stroke in the group of patients with myocardial revascularization compared with optimal medical therapy in patients with chronic coronary heart disease and preserved left ventricular function. “Total events” refers to the sum of all events in the context of stroke, within each study. Risk ratio for each individual study (blue squares), 95% CI (horizontal lines), black diamond (pooled effect size), [11,13,14,16,17,18,19].

The incidence of stroke was reported in seven RCTs [11,13,14,16,17,18,19]. A total of 6682 patients were included in the current study, with 3494 patients assigned to the invasive strategy and 3188 receiving OMT. The mean follow-up duration was 4.3 years. The pooled data analysis results revealed no statistically significant difference in stroke incidence between the two groups (97 cases (2.77%) vs. 72 cases (2.26%); RR: 1.14; 95% CI: 0.85–1.55; *p* = 0.39; I2 = 0%) (Figure 5). This indicates that the study did not find a significant disparity in the effectiveness of invasive and conservative treatment strategies in stroke prevention.

**Figure 6:**
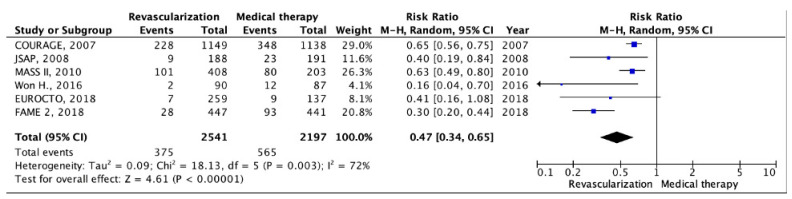
Forest plot of RR for unplanned coronary revascularization in patients undergoing invasive strategy compared with those receiving optimal medical therapy in patients with chronic coronary heart disease and preserved left ventricular function. “Total events” refers to the sum of all events in the context of unplanned coronary revascularization, within each study. Risk ratio for each individual study (blue squares), 95% CI (horizontal lines), black diamond (pooled effect size), [13,14,16,17,18,19].

Data on unplanned coronary revascularization (PCI, CABG, re-intervention, or reoperation) were reported in six RCTs [13,14,16,17,18,19]. The analysis involved a total of 2541 patients in the invasive strategy group and 2197 patients in the OMT group. The mean follow-up duration was 4.1 years. The meta-analysis yielded intriguing results, indicating that patients who underwent myocardial revascularization had a lower incidence of unplanned revascularization procedures (375 cases) compared to patients who received OMT alone (565 cases). Specifically, the rate of unplanned myocardial revascularization was 14.75% in the invasive strategy group, significantly lower than the 25.72% observed in the OMT group (RR: 0.47; 95% CI: 0.34–0.65; *p* < 0.001; I2 = 72%) (Figure 6).

### 5.2. Chronic CHD with LV Systolic Dysfunction

**Figure 7:**
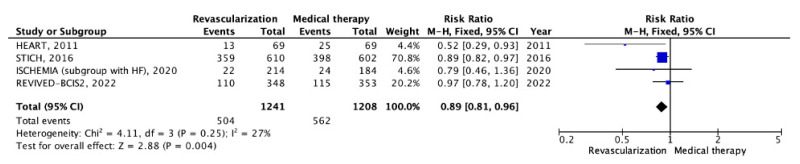
Forest plot of RR for all-cause mortality in the group of patients with chronic coronary heart disease and left ventricular systolic dysfunction depending on myocardial revascularization compared with optimal medical therapy. “Total events” refers to the sum of all events in the context of all-cause mortality, within each study. Risk ratio for each individual study (blue squares), 95% CI (horizontal lines), black diamond (pooled effect size), [10,20,21,22].

We conducted a meta-analysis of outcomes in patients with chronic CHD and LV systolic dysfunction who underwent myocardial revascularization, comparing them with those receiving OMT alone. Four RCTs [10,20,21,22] provided data on the incidence of all-cause mortality. In the myocardial revascularization group, there were 504 events out of 1241 patients, while in the OMT group there were 562 events out of 1208 patients (Table 3). The average follow-up period was 6.6 years. The analysis revealed statistically significant differences in the all-cause mortality rates between the patients undergoing the invasive strategy and those receiving OMT (40.61% vs. 46.52%; RR: 0.89; 95% CI: 0.81–0.96; *p* = 0.004; I2 = 27%) (Figure 7).

**Figure 8:**
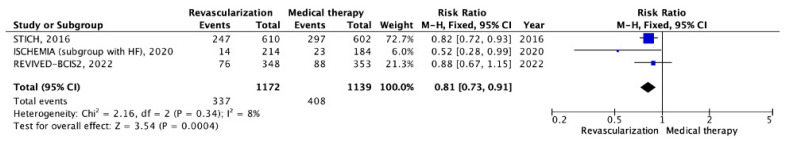
Forest plot of RR for cardiovascular mortality in the group of patients with chronic coronary heart disease and left ventricular systolic dysfunction depending on myocardial revascularization compared with optimal medical therapy. “Total events” refers to the sum of all events in the context of cardiovascular mortality, within each study. Risk ratio for each individual study (blue squares), 95% CI (horizontal lines), black diamond (pooled effect size), [10,16,17,18,21,22].

Three RCTs [10,16,17,18,21,22] provided data on the incidence of cardiovascular mortality and MI. In the myocardial revascularization group, there were a total of 337 events out of 1172 patients, whereas in the OMT group there were 408 events out of 1139 patients. The follow-up period averaged 7.2 years. The meta-analysis revealed a reduced incidence of CV mortality in patients undergoing myocardial revascularization compared to those receiving OMT (28.75% vs. 35.82%; RR: 0.81; 95% CI: 0.73–0.91; *p* = 0.0004; I2 = 0%) (Figure 8).

**Figure 9:**
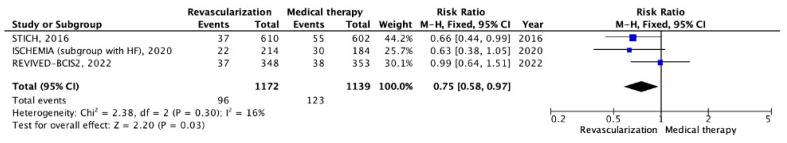
Forest plot of RR for myocardial infarction in the group of patients with chronic coronary heart disease and left ventricular systolic dysfunction depending on myocardial revascularization compared with optimal medical therapy. “Total events” refers to the sum of all events in the context of cardiovascular mortality, within each study. Risk ratio for each individual study (blue squares), 95% CI (horizontal lines), black diamond (pooled effect size), [10,21,22].

A pooled analysis of three RCTs [10,21,22] reported the incidence of spontaneous MI. The analysis included 1172 patients who underwent myocardial revascularization and 1139 patients treated with OMT. The follow-up duration averaged 7.2 years. The group undergoing invasive treatment demonstrated a significantly lower incidence of MI, with 96 cases (8.19%), compared to the patients receiving OMT alone, for which 123 cases (10.8%) were observed (RR: 0.75; 95% CI: 0.58–0.97; *p* = 0.03; I2 = 16%) (Figure 9).

Thus, the meta-analysis revealed no advantages of myocardial revascularization in patients with chronic CHD and preserved LV EF in preventing overall and cardiovascular mortality, as well as in reducing the incidence of MACE. However, patients with LV systolic dysfunction demonstrated a notable improvement in prognosis with the invasive treatment strategy combined with the OMT, leading to reduced mortality and incidence of MI.

## 6. Discussion

In the past decade, despite numerous studies and meta-analyses, the prognostic significance of revascularization in patients with chronic CHD in relation to a reduction in cardiovascular complications remains uncertain.

Numerous large RCTs have demonstrated comparable mortality rates between invasive strategies and conservative therapy [11,13,14,15,16], leading to reduced recommendations for revascularization. This has led to a notable disparity between accumulated clinical experience and the current American College of Cardiology/American Heart Association/Society for Cardiovascular Angiography and Interventions (ACC/AHA/SCAI) clinical guidelines for coronary revascularization (2021), which stipulate that invasive strategies are recommended for patients with multivessel lesions and stenosis of the left anterior descending artery trunk (class IIb). Furthermore, patients with moderate systolic dysfunction and multivessel coronary artery disease do not receive strong recommendations for myocardial revascularization (class IIa) [23]. These findings have prompted our analysis of the treatment strategy choice in chronic CHD based on LV systolic function.

The meta-analysis conducted under the first scenario, which involved a direct comparison of treatment outcomes in patients with preserved LV contractile function, encompassed eight RCTs with a collective population exceeding 13,000 patients. We made a deliberate choice to omit some studies from the analysis, specifically those involving balloon angioplasty [24], a study which compared PCI and exercise but lacked sufficient emphasis on OMT [25], and ISCHEMIA-CKD (2020), which focused on patients with chronic kidney disease [26]. The exclusion of these studies aims to minimize the influence of RCTs conducted in the past, during periods when neither medical therapy nor surgical interventions conformed to the contemporary standards of treatment.

Based on the results obtained, both invasive and conservative strategies demonstrated a comparable reduction in the clinical manifestations of the disease, with no significant differences observed in the achievement of endpoints such as total mortality, death from CV causes, and the occurrence of MI [11,13,14,15,16,17,18,19]. While the incidence of MI was lower in the invasive strategy group compared to the patients receiving OMT, these differences did not reach a statistical significance (9.88% vs. 10.49%, *p* = 0.47). These findings might be attributed to the substantial heterogeneity across these studies (I2 = 61%) and, thus, warrant further investigation. Similar trends were observed in the meta-analysis conducted by Aviral Vij et al. (2021), in which no significant disparities in all-cause mortality were identified between revascularization and OMT. However, revascularization was shown to reduce the incidence of MACE, predominantly by lowering the occurrence of MI by 14% [27].

The absence of a discernible difference between invasive and conservative strategies, as observed in our study and by other researchers, may be accounted for by various hypotheses. For instance, the relatively shorter follow-up period in the group with preserved LV contractile function could explain the initial similarity in outcomes between the studied strategies over the span of four years. However, it is conceivable that a more pronounced divergence in the trajectories of major endpoints could emerge over extended follow-up periods, attributable to a lower incidence of MI and CV mortality in the planned revascularization group. This supposition finds support in the recently published 7-year results of the ISCHEMIA-2023 trial, wherein the revascularization group exhibited a 2.2% lower incidence of CV mortality (6.4% versus 8.6%; adjusted hazard ratio, 0.78 [95% CI, 0.63–0.96]), although no significant difference was noted between strategies in the overall mortality (12.7% in the invasive strategy, 13.4% in the conservative strategy; adjusted hazard ratio, 1.00 [95% CI, 0.85–1.18]) [28].

The absence of a demonstrated advantage in favor of the invasive strategy may also be attributed to the substantial impact of the following RCTs: COURAGE (2007), BARI 2D (2009), and ISCHEMIA (2020) [11,13,15]. The COURAGE (2007) study marked a significant milestone by initially highlighting that there was no discernible disparity between primary myocardial revascularization (OMT, PCI) and OMT. Notably, the use of bare-metal stents was predominant in most PCI as part of the study. While primary revascularization in patients with chronic CHD led to a reduction in the severity of ischemia, these differences were observed to be short-term, and this advantage largely dissipated after a 36-month interval [13]. 

The BARI 2D study (2009) enrolled patients presenting with severe angina pectoris and multivessel coronary lesions combined with type 2 diabetes mellitus [15]. Notably, this RCT stood out due to its randomization process, which hinged on the method of revascularization (PCI or CABG) as determined by the physician responsible. Subsequently, the participants were allocated into a group receiving OMT and those undergoing the selected method of revascularization. Over a span of 5.4 years, no statistically significant disparities were observed in the rates of all-cause mortality between the invasive and conservative approaches. However, it is worth highlighting that the incidence of MACE was markedly lower in the CABG group in comparison to the OMT group (22.4% vs. 30.5%, *p* = 0.002) [15].

The ISCHEMIA study (2020), the largest study, assessed the effectiveness of revascularization (PCI, CABG) versus OMT in patients with moderate to severe ischemia. Importantly, randomization was performed in the absence of coronary angiography outcome data, and second-generation drug-coated stents were used during PCI. Furthermore, a substantial proportion of participants included in the study exhibited either no symptoms of disease (20.1%) or presented with grade II angina pectoris (48.8%) [11]. 

The mid-term findings (3.2 years) from the ISCHEMIA (2020) study revealed that an invasive strategy in the management of chronic CHD is associated with an elevated risk of periprocedural MI and a reduced risk of CV death. Notably, there were no discernible differences in the risk of all-cause mortality, while the risk of non-procedural MI was higher when employing an OMT [11]. Thus, considering the aforementioned factors, it appears that the specific design features of large RCTs, relatively short follow-up durations, and the inclusion of individuals with lower symptom burdens may underlie the absence of definitive benefits associated with primary revascularization in chronic CHD.

Similar findings have been previously reported, as evidenced by the meta-analysis conducted by Bytyci, I. (2023), which indicated that revascularization does not diminish the risk of all-cause and CV mortality, MI, stroke, or hospitalization for angina progression when compared with OMT [29].

However, in contemporary medicine, there remains an insufficiency of RCTs aimed at investigating the ideal treatment approach for chronic CHD in individuals with LV systolic dysfunction. Furthermore, these patients are classified as high-risk and exhibit elevated mortality rates. Hence, our study examined the outcomes derived from a direct comparison of invasive and conservative strategies in patients with chronic CHD and LV systolic dysfunction (the second scenario).

The findings from the meta-analysis of RCTs involving LV systolic dysfunction indicated elevated mortality rates irrespective of the selected strategy. However, we observed a significant decrease in the overall and CV mortality rates when revascularization was performed. Specifically, the rates were 40.61% vs. 46.52% (RR: 0.89; 95% CI: 0.81–0.96; *p* = 0.004) and 28.75% vs. 35.82% (RR: 0.81; 95% CI: 0.73–0.91; *p* = 0.0004), respectively. This confirms the advantages of using an invasive strategy along with OMT for patients with chronic CHD and LV systolic dysfunction. Our analysis of MI incidence also demonstrated the benefits of surgical treatment tactics with rates of 8.19% vs. 10.8% (RR: 0.75; 95% CI: 0.58–0.97; *p* = 0.03). It is important to note, however, that not all studies included in the meta-analysis of LV systolic dysfunction had an adequate number of patients with a high functional class of angina [10,20,21,22].

Three trials, namely HEART (2011), STICH (2016), and REVIVED-BCIS2 (2022), recruited patients with chronic CHD and systolic dysfunction (LV EF < 35%). These patients were randomized into conservative and invasive treatment strategies [20,21,22]. The results from the first 5 years of follow-up in the STICH trial did not show any significant differences in primary outcomes between the two groups. However, it is worth noting that the curves representing the occurrence of events over time intersected at the two-year mark [30]. In the extended version of STICHES, a 16% reduction in the all-cause mortality was observed in patients receiving coronary intervention compared to those receiving OMT after 10 years, thus confirming the benefits of CABG in patients with CAD and significant LV systolic dysfunction [21]. Additional data from the REVIVED-BCIS2 trial (2022) indicated no disparities in the overall mortality and hospitalization rates due to CABG decompensation based on the selected treatment strategy [22]. The lack of a revascularization benefit is likely attributed to performing PCI alone in patients with severe LV systolic dysfunction. Importantly, despite achieving adequate revascularization completeness (71%) in the study, it did not improve disease outcomes [22].

A subgroup analysis of the ISCHEMIA study, specifically focusing on patients with heart failure and LV dysfunction with EF between 35 and 45%, revealed that primary revascularization significantly reduces the incidence of MACE [10]. It is important to note that similar trends were already evident in previous subanalyses when LV EF was lower than 50% (*p* = 0.05) [10].

In the HEART study, despite the small number of patients, a significant difference in the studied strategies was obtained: 25 (37%) patients died in the conservative therapy group, and 26 (38%) patients died in the in the invasive group (*p* > 0.05). However, it is worth mentioning that, among the participants in the primary revascularization group, 22 patients did not undergo the intervention. The comparison between the outcomes of patients treated after revascularization and those receiving OMT demonstrates the advantage of an invasive strategy (37% vs. 29%, respectively, *p* < 0.05) [20].

The disparity in the follow-up periods between the pooled studies for LV systolic dysfunction and preserved LV EF is worth noting, with a longer follow-up period of 6.6 years for the all-cause mortality data and 7.2 years for the CV mortality data in the LV systolic dysfunction group. In comparison, the follow-up periods for preserved LV EF were 4.2 years and 4.0 years, respectively. This contrast highlights the extended prognostic benefits and long-term efficacy of procedural and surgical revascularization techniques in the LV systolic dysfunction group.

Chronic coronary syndrome is recognized for its intricate, multifaceted pathogenesis, attributed not only to obstruction of coronary blood flow, endothelial dysfunction, coronary spasm, and inflammation, but also to microvascular changes, abnormalities in intracellular oxygen transport, and production of mitochondrial energy [31]. Concurrently, it is important to note that medical therapy aimed at inhibiting the progression of atherosclerosis serves as the foremost approach in managing patients with chronic CHD [32].

The low compliance rate among patients undergoing myocardial revascularization and the impact of different factors on adherence to OMT were examined in a meta-analysis conducted by Pinho-Gomes AC et al. (2018) [33].

Even with medical interventions aimed at mitigating adverse outcomes, there exists a cohort of high-risk patients for whom interventional or surgical revascularization continues to showcase its efficacy in lowering mortality. Notably, this is observed predominantly in patients with multivessel coronary lesions, particularly those involving the left coronary artery trunk, as well as individuals with ischemic cardiomyopathy and other severe forms of chronic CHD [23]. Hence, to diminish the incidence of adverse outcomes, it becomes imperative to enhance adherence to OMT, particularly following surgical revascularization.

After conducting an extensive research analysis on LV systolic dysfunction, it was concluded that CABG is the preferred long-term revascularization strategy [21]. Furthermore, according to the meta-analysis by Gaudino M., the employment of CABG not only reduces the overall and cardiovascular mortality but also decreases the occurrence of myocardial infarction and unplanned revascularization when compared to PCI and OMT [34].

According to the meta-analysis conducted by Liga et al. (2023), data have been obtained on the potential benefit of surgical revascularization in patients with chronic CHD and significant depression of LV systolic function [35]. However, the advantage of coronary artery bypass surgery over OMT only becomes significant during the later stages of long-term follow-up. The authors also presented interesting findings, indicating that the assessment of myocardial viability and the extent of induced ischemia do not have an impact on the final results.

Nevertheless, researchers emphasize the importance of revascularization to reduce mortality in patients with heart failure and LV systolic dysfunction. As a result, it is recommended to perform a preliminary assessment of myocardial viability in severe cases of LV systolic dysfunction when determining the need for myocardial revascularization [23,35].

Therefore, this meta-analysis aimed to compare the advantages of invasive and conservative strategies in patients with chronic CHD, taking into consideration the level of LV systolic function. The findings from our study clearly indicate that the choice of treatment approach is influenced not only by the presence of chronic CHD but also by the degree of LV dysfunction. Furthermore, even in cases where surgical treatments do not demonstrate clear advantages in patients with preserved LV function, there is still a noteworthy decrease in the incidence of unplanned revascularizations.

## 7. Limitations

The results of our meta-analysis must be cautiously interpreted in light of several limitations. A common feature of studies examining treatment outcomes for chronic CHD is the exclusion of patients with reduced LV EF, resulting in insufficient data to draw conclusions. There is a scarcity of studies on the selection of effective treatment strategies for individuals with ischemic LV systolic dysfunction, which explains the limited number of RCTs included in our meta-analysis.

The overall analysis included patients with reduced LV EF ranging from 27% to 45%, which might have impacted the obtained results. Due to the limited number of studies, it is not feasible to conduct a more comprehensive analysis of the influence of myocardial contractility on the optimal choice of treatment strategies [10,20,21,22].

To obtain data on the incidence of endpoints for those with preserved LV function from ISCHEMIA study we subtracted the results of the studied subgroup with heart failure or LV dysfunction [10] from those of the overall ISCHEMIA trial [11]. However, it is important to note that our analysis was limited by the unavailability of patient-specific information. Therefore, we were unable to differentiate between periprocedural and spontaneous MI.

Some of the studies included in the meta-analysis have limitations. For instance, they included low-risk patients with functional class I–II angina pectoris (JSAP, ISCHEMIA), employed bare metal stents or stents with first-generation drug-eluting agents (COURAGE, JSAP, BARI 2D, MASS II), and conducted randomization with known coronary anatomy [13,14,15,16]. Moreover, the majority of studies included in the meta-analysis lacked risk stratification of adverse events based on the mode of revascularization. In the FAME 2 study, 12.7% of patients with LV EF < 50% were included [19]. Additionally, not all studies provided information regarding the severity of myocardial ischemia, concurrent pathology, and disease outcomes. Consequently, definitive conclusions about the rationale behind selecting one strategy over another cannot be made.

## 8. Conclusions

The current meta-analysis, which included studies involving patients with chronic CHD, has demonstrated that the effectiveness of the selected strategy depends on the contractility of the LV. Importantly, when revascularization is added to OMT in individuals with preserved LV EF, there are no noticeable advantages in terms of reducing the overall mortality, CV mortality, or MACE rates. However, among patients who underwent primary myocardial revascularization, the need for repeat procedures was lower compared to those who received OMT alone. 

It was found that combining revascularization with OMT led to reduced overall mortality, CV mortality, and the need for unplanned revascularization in patients with chronic CHD and LV systolic dysfunction.

To establish conclusive findings, additional evidence is crucial to fully explore the most effective treatment strategy for patients with chronic CHD and LV systolic dysfunction. In future investigations, patients should be properly randomized to ensure unbiased results. Additionally, objective examination methods should be employed to assess not only the extent of coronary lesions and the presence of disease symptoms but also to evaluate heart failure by categorizing patients based on the degree of LV EF reduction. Furthermore, it is imperative to assess the frequency of primary and secondary outcomes based on the method of myocardial revascularization in individuals with chronic CHD.

## Figures and Tables

**Table 1:** Overview of the studies included in the systematic review.

Trials, Year Published (Region)	Study Years/Follow-Up	Study Group	Strategy Being Studied	Primary Endpoints	Secondary Endpoints	Number of Patients (*n*)
COURAGE, 2007 (North America), [13]	1999–2004/ 4.6	Chronic CHD, stenosis ≥70% in at least one proximal epicardial coronary artery and objective evidence of myocardial ischemia or at least one coronary stenosis of ≥80% and classic angina without provocative testing	OMT, PCI	Death from any cause and nonfatal myocardial infarction	Composite of death, MI, stroke, and hospitalization for unstable angina with negative biomarkers, quality of life	2287
JSAP, 2008 (Japan), [14]	2002–2004/ 3.3	Chronic CHD low-risk consisting of one- or two-vessel disease, stenosis ≥75%, and objective evidence of myocardial ischemia	OMT, PCI	Death (total death, cardiac death, and sudden death), acute coronary syndrome (MI or UAP), CVA (cerebral infarction or cerebral hemorrhage), and emergency hospitalization.	Evaluation of the angina severity grade 1 month, 6 months, 1 year, 2 years, and 3 years after registration and elective repeat revascularization.	384
BARI 2D, 2009 (USA, Europe), [15]	2001–2005/ 5.3	Both type 2 diabetes and coronary artery disease, ≥50% stenosis of a major epicardial coronary artery associated with a positive stress test or ≥70% stenosis of a major epicardial coronary artery and classic angina	OMT, PCI, CABG	Death from any cause	Composite of death, MI, or stroke	2368
MASS II, 2010 (Brazil), [16]	1995–2000/ 10	Chronic CHD, multivessel coronary stenosis of more than 70% by means of visual assessment and documented ischemia (class II or III)	OMT, PCI, CABG	Overall death, MI, and angina that required mechanical revascularization	Angina status, death due to a cardiac cause, and a cerebrovascular accident.	611
HEART, 2011 (United Kingdom), [20]	2001–2004/ 4.9	Heart failure, coronary artery disease, and LV EF < 35%, which had at least five viable segments with reduced contractility	OMT, PCI, CABG	Death from any cause	-	138
Won H., 2016 (Republic of Korea), [17]	2010–2012/ 1	Chronic CHD, stenosis in at least one proximal epicardial coronary artery (diameter stenosis of ≥70%)	OMT, PCI	Death from any cause, MI, stroke, repeat revascularization.	-	177
STICH, 2016 (USA, Canada, Europe), [21]	2002–2007/ 10	Chronic CHD that was amenable to CABG and LV EF of 35% or lower	OMT, CABG	Death from any cause	Death from cardiovascular causes, death from any cause or hospitalization for cardiovascular causes, death from any cause or hospitalization for heart failure, death from any cause or hospitalization for any cause, and death from any cause or revascularization.	1212
FAME 2, 2018 (Europe, North America), [19]	2010–2012/ 5	Chronic angina or documented silent ischemia that had at least one stenosis with 50% of its diameter in a large epicardial artery	OMT, PCI	Composite of death from any cause, MI, or urgent revascularization	Components of the primary endpoint as well as death from cardiac causes, any revascularization, stroke, and stent thrombosis	888
EUROCTO, 2018 (France), [18]	2012–2015/ 1	Chronic CHD, angina + ≥1 chronic coronary total occlusions	OMT, PCI	Change in health status subscales as assessed by SAQ	Changes from baseline to 12 months of EQ-5D and the Canadian Cardiology Society classification, and major cardiac adverse events, stent thrombosis, cerebrovascular events, and hospitalization for cardiac reasons	396
ISCHEMIA, 2020 (USA), [11]	2012–2018/ 3.3	Chronic CHD, stress testing showed moderate or severe reversible ischemia on imaging tests or severe ischemia on exercise tests without imaging	OMT, PCI, CABG	Composite of death from cardiovascular causes, MI, or hospitalization for unstable angina, heart failure, or resuscitated cardiac arrest	Composite of death from cardiovascular causes or MI and angina-related quality of life.	5179
ISCHEMIA, left ventricular dysfunction, 2020 (USA), [10]	2012–2018/ 3.2	Chronic CHD, LV EF 35–45%	OMT, PCI, CABG	Composite of cardiovascular death, MI, resuscitated cardiac arrest, or hospitalization for unstable angina or heart failure	All-cause death, cardiovascular death, MI, hospitalization for UAP, hospitalization for heart failure	398
REVIVED-BCIS2, 2022 (United Kingdom), [22]	2013–2020/ 8.5	Chronic CHD, multivessel coronary stenosis, LV EF of 35% or less	OMT, PCI	Composite outcome was death from any cause or hospitalization for heart failure	Components of the primary outcome, death from cardiovascular causes, appropriate ICD therapy (antitachycardia pacing or shocks, or both, for either ventricular tachycardia or ventricular fibrillation), MI, unplanned revascularization, serial NT-proBNP levels, the Canadian Cardiovascular Society angina class, and major bleeding	700

CHD—chronic coronary heart disease; OMT—optimal medical therapy; PCI—percutaneous coronary intervention; CABG—coronary artery bypass grafting; UAP—unstable angina pectoris; MI—myocardial infarction; LV EF—left ventricular ejection fraction; CVA—cerebrovascular accidents; NT-proBNP—N-terminal pro-B-type natriuretic peptide; ICD—implantable cardioverter defibrillator. Data presented: *n* (%).

**Table 2:** Main endpoints of studies included in a systematic review comparing treatment strategies in patients with chronic CHD with preserved LV EF.

Name Research, Year	All-Cause Mortality		Cardiovascular Death		Myocardial Infarction		Cerebrovascular Accidents		Unplanned Revascularization	
	OMT	INV	OMT	INV	OMT	INV	OMT	INV	OMT	INV
COURAGE, 2007, [13]	95 (8.3)	85 (7.6)	25	23	128 (12.3)	143 (13.2)	14 (1.8)	22 (2.1)	348 (32.6)	228 (21.1)
JSAP, 2008, [14]	7 (3.9)	6 (2.9)	3	2	7 (3.8)	3 (1.6)	2 (1.1)	2 (0.6)	23 (11.7)	9 (5.0)
BARI 2D, 2009, [15]	161 (13.5)	155 (13.2)	nd	nd	138 (11.6)	118 (10)	33 (2.8)	30 (2.6)	-	-
MASS II, 2010, [16]	63 (31)	PCI—49 (24.1) CABG—51 (25.1)	42 (20.7)	PCI—29 (14.3) CABG—22 (10.8)	42 (20.7)	PCI—27 (13.3) CABG—20 (10.3)	14 (6.9)	PCI—11 (5.4) CABG—17 (8.4)	80 (39.4)	PCI—86 (41.9) CABG—15 (17.4)
Won H., 2016, [17]	4 (4.6)	6 (6.7)	2 (2.3)	1 (1.1)	3 (3.4)	1 (1.1)	1 (1.1)	1 (1.1)	12 (13.8)	2 (2.2)
FAME 2, 2018, [19]	23 (5.2)	23 (5.1)	7 (1.6)	11 (2.5)	53 (12.0)	36 (8.1)	7 (1.6)	12 (2.7)	93 (21.1)	28 (6.3)
EUROCTO, 2018, [18]	0	2 (0.8)	0	2 (0.8)	0	5 (1.9)	1 (0.7)	2 (0.8)	9 (6.7)	7 (2.9)
ISCHEMIA, 2020, [11]	144 (5.6)	145 (5.6)	111	92	233 (9.0)	210 (8.1)	38	45	nd	nd

CHD—chronic coronary heart disease; OMT—optimal medical therapy; INV—invasive strategy; LV EF—left ventricular ejection fraction; CABG—coronary artery bypass grafting; PCI—percutaneous coronary intervention; nd—no data. Data presented: *n* (%).

**Table 3:** Main endpoints of studies included in a systematic review comparing treatment strategies in patients with chronic CHD and LV systolic dysfunction.

Name Research, Year	All-Cause Mortality		Cardiovascular Death		Myocardial Infarction		Cerebrovascular Accidents		Unplanned Revascularization	
	OMT	INV	OMT	INV	OMT	INV	OMT	INV	OMT	INV
HEART, 2011, [20]	25 (37)	13 (29)	nd	nd	nd	nd	nd	nd	nd	nd
STICH, 2016, [21]	398 (66.1)	359 (58.9)	297 (49.3)	247 (40.5)	55 (9.1)	37 (6.1)	41 (6.8)	47 (7.7)	50 (8.3)	43 (7.0)
ISCHEMIA, left ventricular dysfunction, 2020 [10]	24 (13.3)	22 (10.2)	23 (12.7)	14 (6.7)	30 (16.5)	22 (10.5)	nd	nd	nd	nd
REVIVED-BCIS2, 2022 [22]	115 (32.6)	110 (31.7)	88 (24.9)	76 (21.9)	38 (10.8)	37 (10.7)	nd	nd	37 (10.5)	10 (2.9)

CHD—chronic coronary heart disease; LV—left ventricular; OMT—optimal medical therapy; INV—invasive strategy; nd—no data. Data presented: *n* (%).

## Supplementary Materials

The following supporting information can be downloaded at https://www.mdpi.com/article/10.3390/pathophysiology30040046/s1: Figure S1: Funnel plots for estimation of systematic bias; Table S1: Assessment of risk of bias in the included studies using RoB2 for RCTs studies; Table S2: Clinical characteristics of the patients.
