# Supplementary material for: Treatment Strategies for Chronic Coronary Heart Disease with Left Ventricular Systolic Dysfunction or Preserved Ejection Fraction—A Systematic Review and Meta-Analysis
Source: Pathophysiology. 2023 Dec 18;30(4):640–58. doi: 10.3390/pathophysiology30040046 (PMC10747738; doi:10.3390/pathophysiology30040046)
Supplement: Supplementary file 1 [file pathophysiology-30-00046-s001.zip › pathophysiology-2760014-supplementary.pdf]

Supplementary Materials

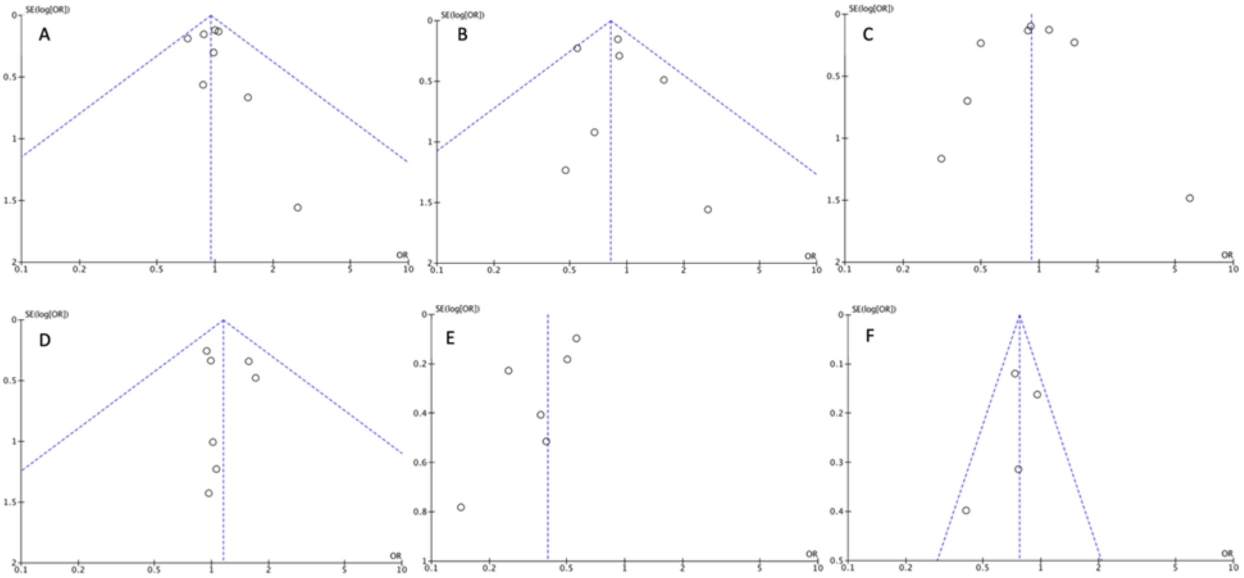

Figure S1. Funnel plots for estimation of systematic bias.
A - All-cause mortality; B - cardiovascular death; C - Myocardial Infarction; D - Stroke; E – Unplanned coronary revascularization; F - All-cause mortality (patients with left ventricular systolic dysfunction).

Table S1. Assessment of risk of bias in the included studies using RoB2 for RCTs studies.

Study, year	Randomization process	Deviation from intervention	Missing outcome data	Measurement of the outcome	Selection of the results	Overall
COURAGE, 2007	L	L	L	L	L	L
JSAP, 2008	L	L	L	L	L	L
BARI 2D, 2009	L	L	L	L	S	S
MASS II, 2010	L	L	L	L	L	L
HEART, 2011	L	L	L	L	S	S
Won H., 2016	L	L	L	L	L	L
STICH, 2016	L	L	L	L	L	L
FAME 2, 2018	L	L	L	L	L	L
EUROCTO, 2018	L	S	L	L	L	S
ISCHEMIA, 2020	L	L	L	L	L	L
REVIVED-BCIS2, 2022	L	L	L	L	L	L

** - L: low; S: some concerns; H: high, RCTs - randomized clinical trials*

Overall risk-of-bias judgment

Criteria

Low risk of bias - The study is judged to be at low risk of bias for all domains for this result.

Some concerns - The study is judged to raise some concerns in at least one domain for this result, but not to be at high risk of bias for any domain.

High risk of bias - The study is judged to be at high risk of bias in at least one domain for this result. The study is judged to have some concerns for multiple domains in a way that substantially lowers confidence in the result.

Table S2. **Clinical characteristics of the patients.**

**Name research, year**	**Age, years**		**Body-mass index, kg/m²**		**Hypertension, no. (%)**		**Diabetes, no. (%)**		**Previous myocardial infarction, no. (%)**		**Cerebrovascular disease, no. (%)**		**Renal insufficiency, no. (%)**		**Peripheral vascular disease, no. (%)**		**Left ventricular ejection fraction, %**	
	**OMT**	**INV**	**OMT**	**INV**	**OMT**	**INV**	**OMT**	**INV**	**OMT**	**INV**	**OMT**	**INV**	**OMT**	**INV**	**OMT**	**INV**	**OMT**	**INV**
COURAGE, 2007	61.8± 9.7	61.5± 10.1	28.7± 0.18	28.5± 0.19	764 (67)	757 (66)	399 (35)	367 (32)	439 (39)	437 (38)	102 (9)	100 (9)	nd	nd	nd	nd	60.9± 10.3	60.8± 11.2
JSAP, 2008	64.2± 7.6	64.5± 7.2	24.1± 2.8	24.3± 3.3	121 (63.4)	119 (63.3)	76 (39.8)	76 (40.4)	28 (15.1)	25 (14.0)	10 (5.4)	13 (7.3)	nd	nd	nd	nd	65.8± 9.6	64.0± 9.7
BARI 2D, 2009	62.4± 9.0	62.3± 8.8	32.2± 6.2	32.0± 6.3	nd	nd	991 (100)	953 (100)	(32.4)	(31.7)	119 (10)	111 (9.5)	nd	nd	(23.7)	(23.7)	57.3± 10.7	57.0± 11.3
MASS II, 2010	60.0± 9.0	60.0± 9.0	nd	nd	111 (55)	252 (61,8)	73 (36)	106 (26)	79 (39)	189 (46)	nd	nd	nd	nd	nd	nd	68.0± 7.0	67.0± 8.0
HEART, 2011	69 (60-74)	65 (58-70)	27,4 (24,2-31,5)	27,5 (24,8-32,0)	34 (49)	35 (51)	23 (33)	28 (41)	50 (73)	51 (74)	8 (12)	12 (17)	nd	nd	11 (16)	16 (23)	LVEF<35%	
Won H., 2016	78.3±4.7	78.0±4.2	nd	nd	65 (74.7)	74 (82.2)	28 (32.2)	36 (40.0)	4 (4.6)	6 (6.7)	-	-	0	2 (2.2)	nd	nd	LVEF≥45%	
STICH, 2016	59 (53-67)	60 (54-68)	27 (24-30)	27 (24-30)	370 (61)	358 (59)	238 (40)	240 (39)	472 (78)	462 (76)	41 (7)	51 (8)	45 (7)	49 (8)	nd	nd	28 (21-34)	27 (22-33)
EUROCTO, 2018	64.7± 9.9	65.2± 9.7	28.3± 5.2	28.4± 4.9	98 (71.5)	189 (73.0)	40 (29.2)	85 (32.8)	25 (18.3)	59 (22.8)	nd	nd	nd	nd	nd	nd	55.7± 10.8	54.5± 10.8
FAME 2, 2018	63.9± 9.6	63.5± 9.4	28.4± 4.6	28.3± 4.3	343 (77.8)	347 (77.6)	117 (26.5)	123 (27.5)	165 (37.4)	164 (36.7)	28 (6.3)	33 (7.4)	12 (2.7)	8 (1.8)	47 (10.7)	43 (9.6)	LVEF≥50%	

ISCHEMIA, 2020	64 (58-70)	64 (58-70)	nd	nd	1895 (73.4)	1894 (73.4)	1071 (42.2)	1093 (41.4)	496 (19.2)	495 (19.2)	176 (6.8)	201 (7.8)	nd	nd	88 (3.4)	116 (4.5)	60 (55-65)	60 (55-65)
ISCHEMIA, left ventricular dysfunction, 2020	64 (57.71)		nd	nd	167 (76.6)		106 (48.0)		77 (35.2)		nd	nd	nd	nd	10 (4.5)		35-45	
REVIVED-BCIS2, 2022	68.8± 9.1	70.0± 9.0	28.7± 5.4	28,4± 5.5	207 (59)	184 (53)	153 (43)	136 (39)	197 (56)	175 (50)	46 (13)	38 (11)	nd	nd	46 (13)	48 (14)	27.0± 6.9	27.0± 6.6

* - OMT - optimal medical therapy; INV - invasive strategy; nd - no data.
